# Supplementary figures and images for: The salivary protein Saglin facilitates efficient midgut colonization of Anopheles mosquitoes by malaria parasites
Source: PLoS Pathog. 2023 Mar 2;19(3):e1010538. doi: 10.1371/journal.ppat.1010538 (PMC10013899; doi:10.1371/journal.ppat.1010538)

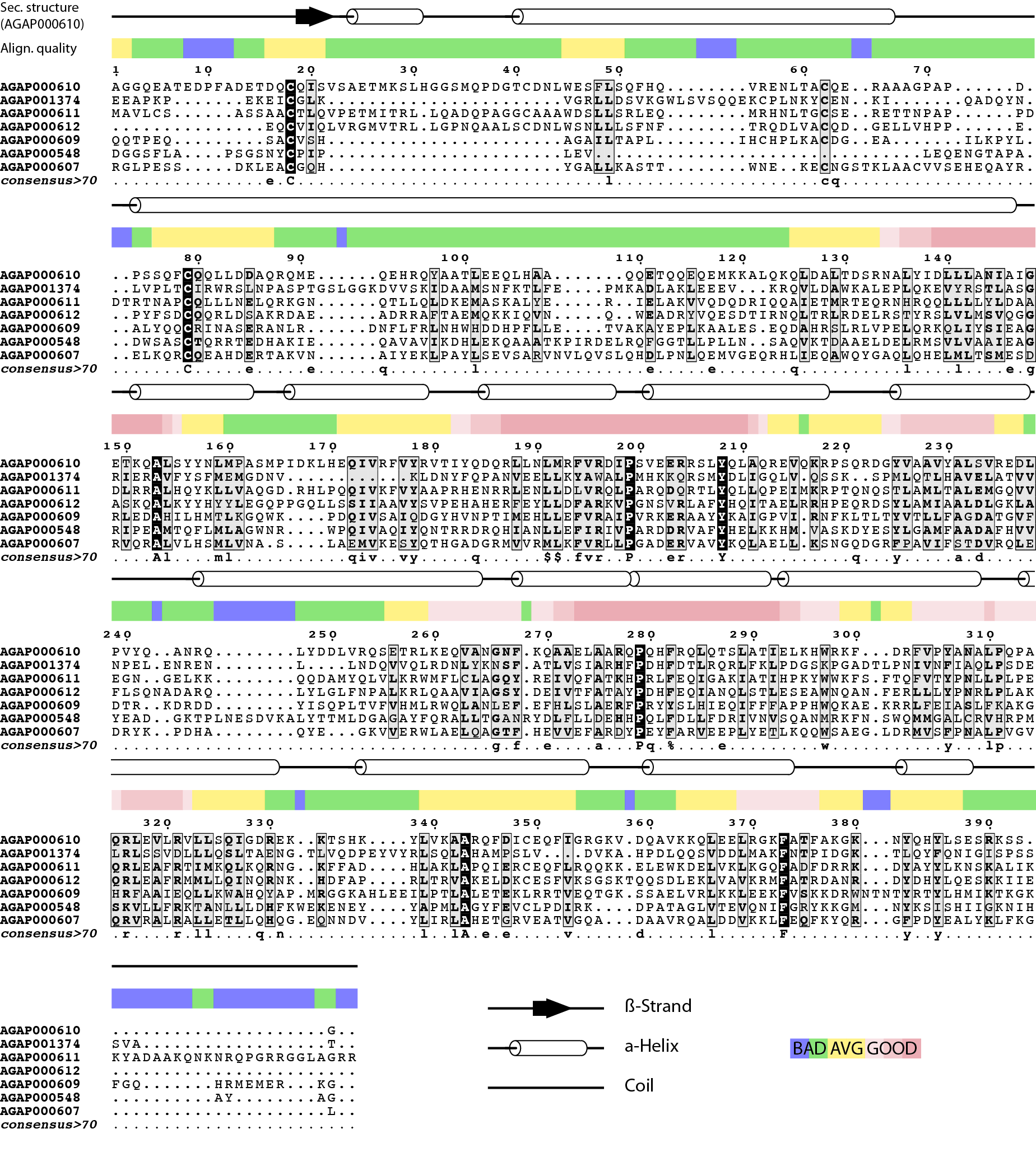

Supplement: S1 Fig — The initial alignment was performed with PSI-Coffee [55] while sequence similarities based on physico-chemical properties of amino acids were calculated with ESPript [56]. The colored stripe above the alignment indicates the quality of the alignment according to ESPrit. In addition the secondary structure of Saglin (AGAP000610) predicted by I-Tasser is shown along the alignment [51]. Please note that the signal peptides from all proteins except AGAP000611, for which no signal peptide has been predicted, were removed in preparation of the alignment. (TIF) [file ppat.1010538.s001.tif]

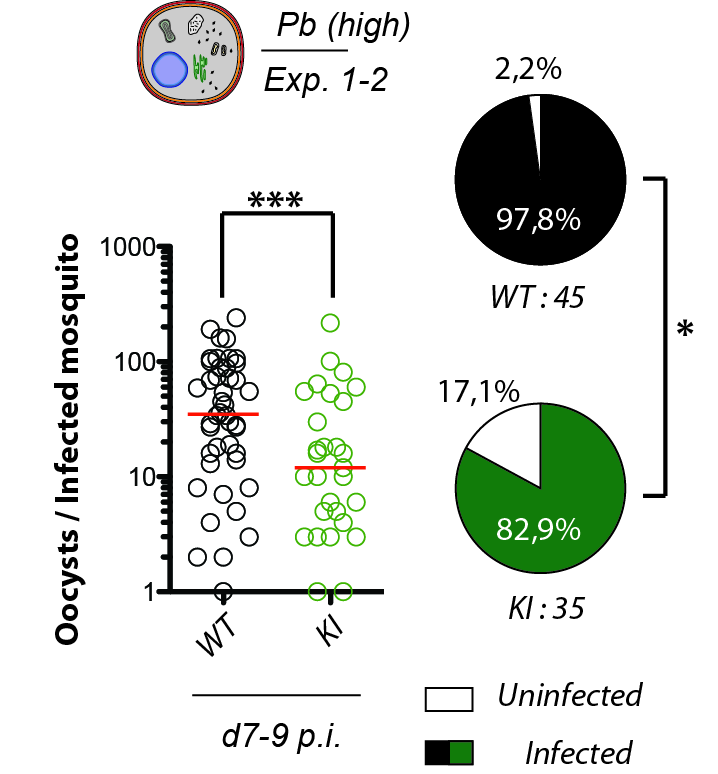

Supplement: S2 Fig — Oocyst densities in sag(-)KI and wild-type (Ngousso) mosquitoes derived from two different colonies. Results of two pooled experiments. The red lines indicate medians, comparison using Mann Whitney test: ***p = 0.0007. Pie charts represent prevalence of infection. Fisher’s exact test: *p = 0.0394. (TIF) [file ppat.1010538.s002.tif]

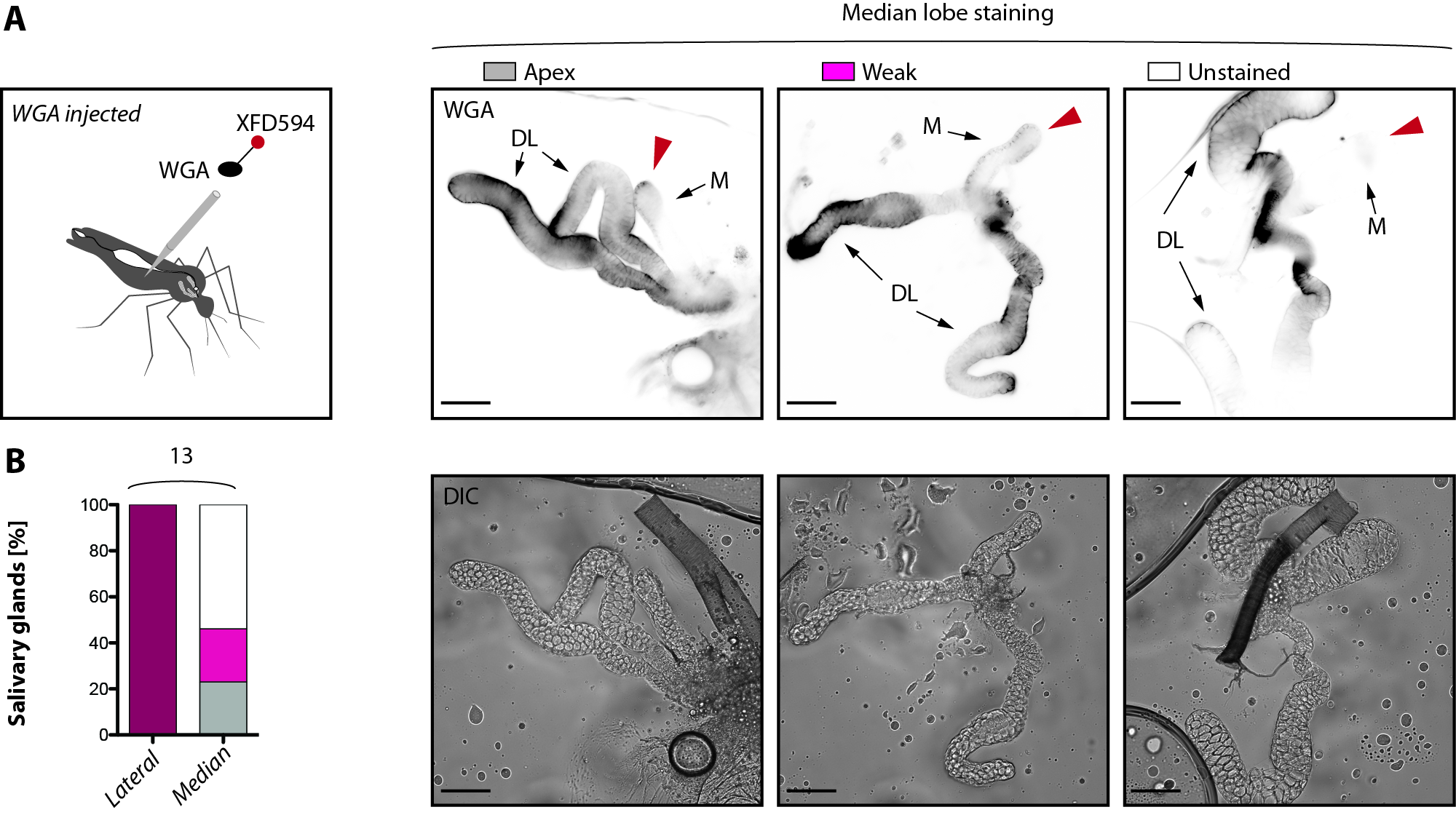

Supplement: S3 Fig — A) Staining pattern of SGS in mosquitoes injected with wheat germ agglutinin (WGA) coupled to XFD594. WGA-XFDS594 was injected into living mosquitoes using a capillary and mosquitoes were dissected one hour after injection. Lateral lobes (both distal DL and proximal, PL) were stained in all samples. Staining patterns in median lobes (M) were classified as „apex“, „weak”and „unstained”. Images illustrating each pattern are given showing localisation of WGA-XFD594 (top) and differential interference contrast (DIC, bottom). Scale bar: 100 μm. B) Quantification of staining patterns according to (A). Thirteen salivary glands were analyzed. (TIF) [file ppat.1010538.s003.tif]

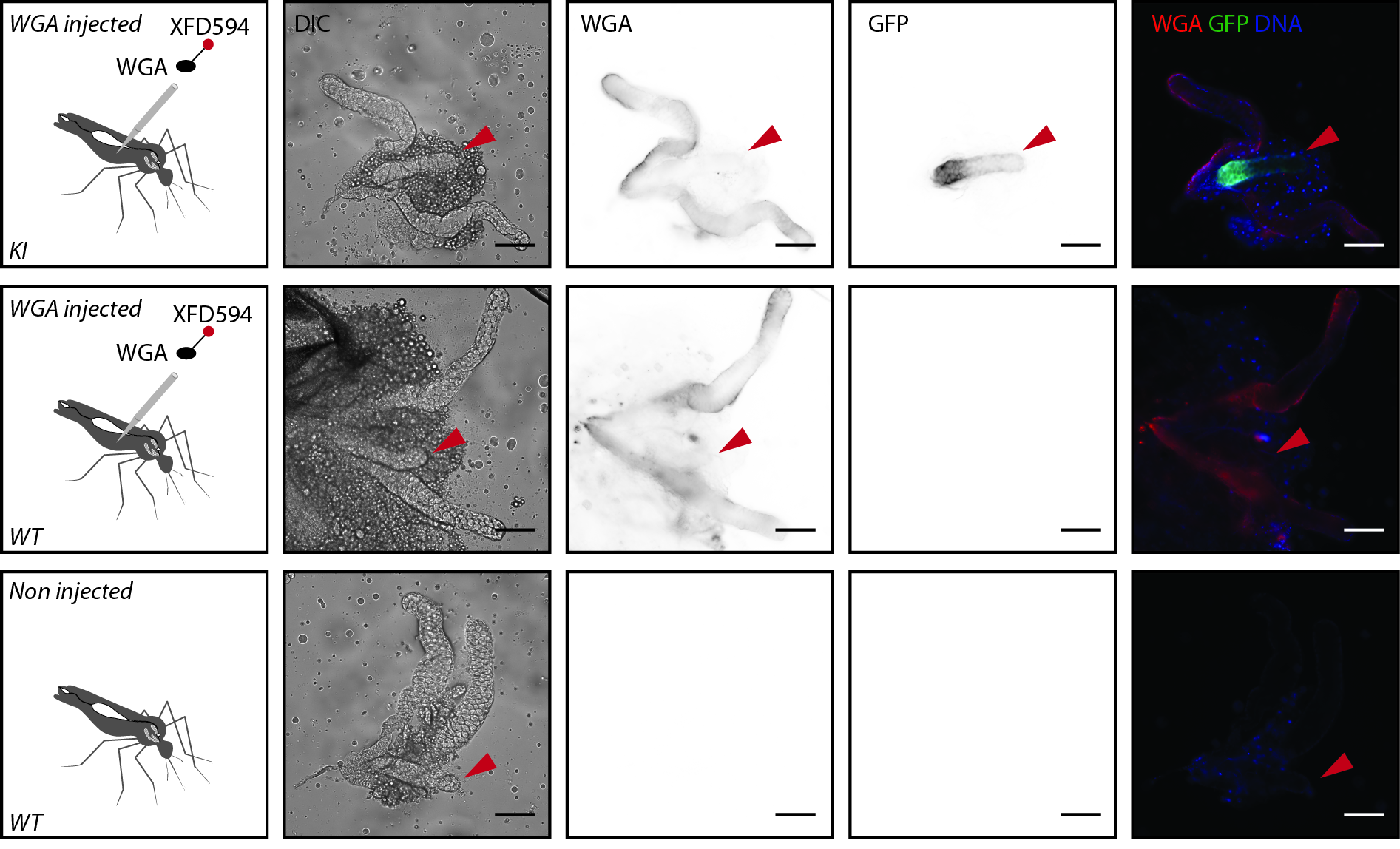

Supplement: S4 Fig — Staining patterns of salivary glands dissected from female mosquitoes injected with wheat germ agglutinin (WGA) coupled to XFD594. The salivary gland staining of an injected female homozygous for sag(-)KI is compared to two salivary glands dissected from an injected and a non-injected wild-type (control) female. Scale bar: 100 μm. Columns from left to right: mosquitoes genotypes, differential interference contrast (DIC), WGA and GFP signal in black on white; merge of WGA (red), GFP (green) and nucleic acid staining (DNA). Red arrowheads indicate median lobes. (TIF) [file ppat.1010538.s004.tif]

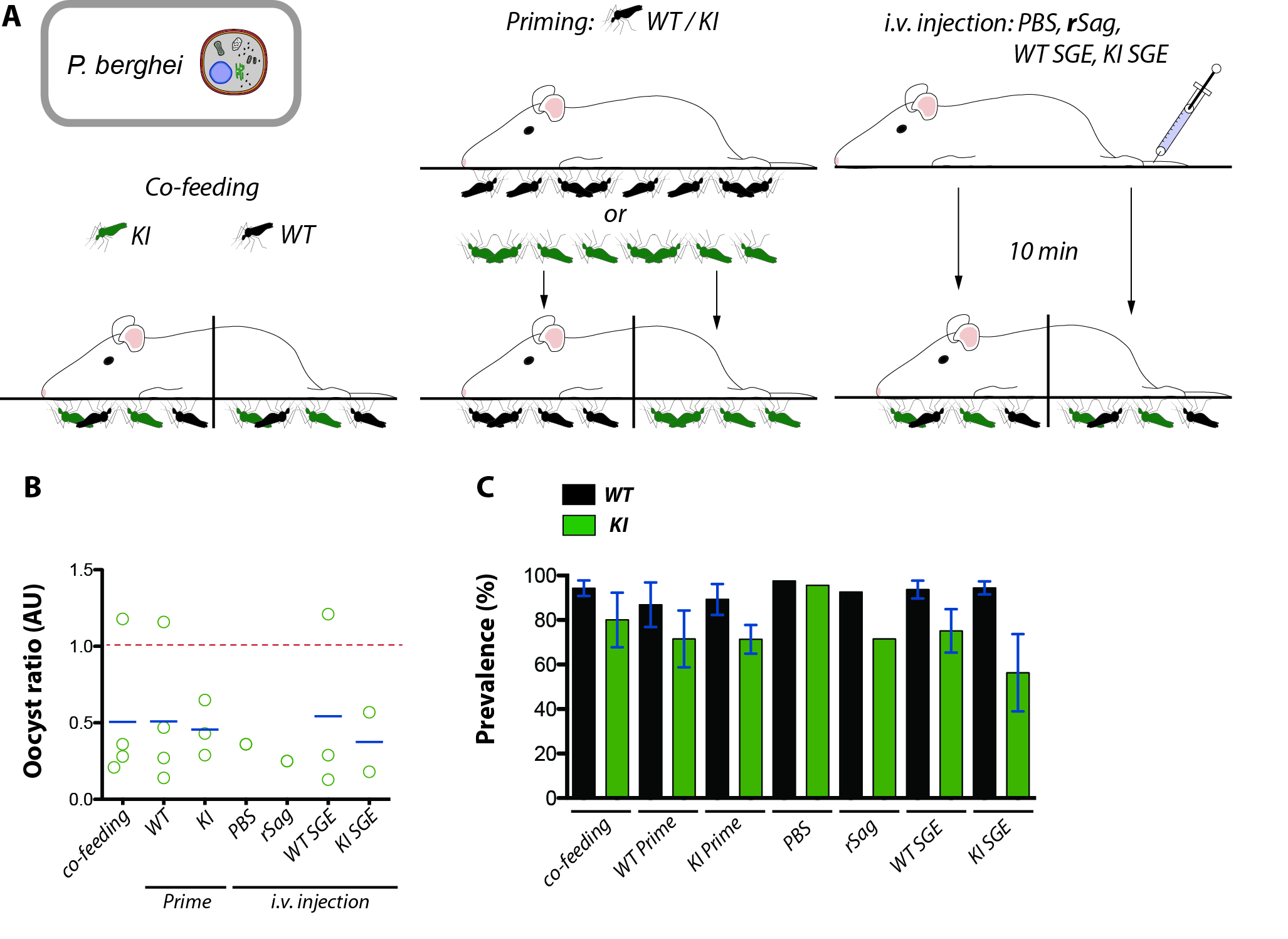

Supplement: S5 Fig — A) Co-feeding of sag(-)KI (KI) and wild-type (WT) mosquitoes on the same P. berghei infected mouse, infections of sag(-)KI and wild-type mosquitoes on mice “primed” with wild-type and sag(-)KI and intravenous (i.v.) injection of PBS, recombinant Saglin (rSag) and salivary gland extracts. B) Each dot represents one experiment, 1–4 independent experiments for each group. Solid blue lines indicate the means, and the dashed blue line a ratio of 1 (expected if the sag(-)KI phenotype is rescued). C) Prevalence of infection for experiments shown in B. Shown is the mean with the standard error of the mean (SEM) in blue. (TIF) [file ppat.1010538.s005.tif]
