# Supplementary material for: The salivary protein Saglin facilitates efficient midgut colonization of Anopheles mosquitoes by malaria parasites
Source: PLoS Pathog. 2023 Mar 2;19(3):e1010538. doi: 10.1371/journal.ppat.1010538 (PMC10013899; doi:10.1371/journal.ppat.1010538)
Supplement: Attachment — Submitted filename: PPATHOGENS-D-22-00735_rebuttal_letter_071222_141222.docx [file ppat.1010538.s006.docx]

Dear Mr. Klug,

Thank you very much for submitting your manuscript "The salivary protein Saglin facilitates efficient midgut colonization of Anopheles mosquitoes by malaria parasites" for consideration at PLOS Pathogens. As with all papers reviewed by the journal, your manuscript was reviewed by members of the editorial board and by several independent reviewers. In light of the reviews (below this email), we would like to invite the resubmission of a significantly-revised version that takes into account the reviewers' comments.

Reviewers 1 and 2 both criticize the lack of direct evidence that ingested saglin affects parasite development in the midgut. Addressing this point with additional data would help support the most novel aspect of your work and increase its impact significantly. The obvious experiment would be to ask if your recombinant saglin, if added to an infectious feed or otherwise applied to the gut, complements the saglin knockout. We would consider such direct evidence important to support your conclusion that saglin must be re-ingested to affect the parasite. In the absence of additional evidence, or if the data do not support your proposed mechanism, please phrase your statement in the abstract and elsewhere more carefully.

We would like to thank the three reviewers for their interesting comments and suggestions. Please note that the line numbers given below always refer to the manuscript file without tracked changes. As suggested, we performed additional experiments to rescue the *sag(-)KI* phenotype. Unfortunately, we did not find a clear effect. We now discuss possible reasons for these results in detail (lines 739-754) and in the comments section of Reviewer 1. In agreement with your suggestions, we have made changes in the abstract and throughout the manuscript to avoid over-interpretation of data. We believe we have worded very carefully and can substantiate our conclusions based on the existing data.

Reviewer 1 also raises the question of the mechanism by which saglin affects oocyst numbers, which you cover in your discussion. Further insights would increase the impact of the manuscript, but we would consider this outside the scope of the current analysis.

This is a very important question to follow up, although indeed, not in this manuscript. We have detailed how we addressed this comment below, in our response to Reviewer 1.

Please pay special attention to the summary section of Reviewer 3. While this reviewer gets the expected mass of mosquitoes wrong, they raise important methodological concerns that we ask you to consider and address carefully. Uncertainties and variance in measurements should be noted and propagated through calculations where appropriate.

We have carefully considered the comments of Reviewer 3 below and addressed potential concerns (see below). Most of his comments are due to minor errors in the figure legends (e.g. **Fig. 3B**) as well as in the M&M. We have corrected these errors and added missing information wherever relevant.

Fig. 3B is said to show “Numbers of exflagellated male microgametes” but the axis label reads “microgametocytaemia (%)”. What is it, and if it is the former, what is the unit?

Indeed, there was a mistake in the figure legend. This figure shows the percentage of observed exflagellated microgametes in relation to the number of red blood cells. We corrected the legend in the revised manuscript (lines 1.023-1.024).

We cannot make any decision about publication until we have seen the revised manuscript and your response to the reviewers' comments. Your revised manuscript is also likely to be sent to reviewers for further evaluation.

When you are ready to resubmit, please upload the following:

[1] A letter containing a detailed list of your responses to the review comments and a description of the changes you have made in the manuscript. Please note while forming your response, if your article is accepted, you may have the opportunity to make the peer review history publicly available. The record will include editor decision letters (with reviews) and your responses to reviewer comments. If eligible, we will contact you to opt in or out.

[2] Two versions of the revised manuscript: one with either highlights or tracked changes denoting where the text has been changed; the other a clean version (uploaded as the manuscript file).

Important additional instructions are given below your reviewer comments.

Please prepare and submit your revised manuscript within 60 days. If you anticipate any delay, please let us know the expected resubmission date by replying to this email. Please note that revised manuscripts received after the 60-day due date may require evaluation and peer review similar to newly submitted manuscripts.

Thank you again for your submission. We hope that our editorial process has been constructive so far, and we welcome your feedback at any time. Please don't hesitate to contact us if you have any questions or comments.

Sincerely,

Oliver Billker

Associate Editor

PLOS Pathogens

Kirk Deitsch

Section Editor

PLOS Pathogens

Kasturi Haldar

Editor-in-Chief

PLOS Pathogens

orcid.org/0000-0001-5065-158X

Michael Malim

Editor-in-Chief

PLOS Pathogens

orcid.org/0000-0002-7699-2064

***********************

Reviewer's Responses to Questions

Part I - Summary

Please use this section to discuss strengths/weaknesses of study, novelty/significance, general execution and scholarship.

Reviewer #1: In this study, Klug et al revisit the role of Saglin during Plasmodium infection in mosquitoes. Saglin has been previously identified as a key protein required for colonization of A. gambiae salivary glands by P. falciparum sporozoites, acting as a receptor for the parasite protein TRAP (Ghosh et al Plos Pathogens 2009). However, a more recent study showed that saglin is expressed mainly in salivary gland median (but not lateral) lobes and that induction of saglin expression in lateral lobes does not influence P. falciparum infection in transgenic A. stephensi mosquitoes, questioning the role of saglin (O’Brochta et al Malaria Journal 2019).

Here the authors generated saglin knockout A. gambiae mosquitoes. The absence of saglin caused a decrease in P. berghei and P. falciparum oocyst numbers, but no specific effect on salivary gland colonization by sporozoites. The authors found that knockout of saglin has no effect on mosquito blood feeding, but is associated with reduced numbers of male gametes and traversing ookinetes in the midgut. This translates into reduced parasite transmission at low infection densities in the P. berghei-mouse model. The authors analyzed saglin expression pattern, based on promoter activity and immunofluorescence assays, revealing that the protein is female-specific and restricted to the median lobes of the salivary glands, consistent with previous observations (O’Brochta et al Malaria Journal 2019). They also report that saglin is excreted in the saliva and re-ingested with the blood meal during blood feeding. Based on these observations, they propose that saglin ingested with the blood meal may promote infection in the midgut. Finally, the data show that saglin knockout has no fitness cost in mosquitoes, indicating that this gene could be a potential candidate for gene drive strategies.

The manuscript is well written and the work is very well executed. The data provide compelling genetic evidence ruling out a role for saglin during colonization of the salivary gland by Plasmodium sporozoites. Interestingly, the data also indicate that saglin plays a role at an earlier step, during infection of the mosquito midgut. The authors propose that saglin present in the saliva is re-ingested during the blood meal and may promote establishment of infection in the midgut. This is an interesting model and the main novelty here. However, this part of the study would benefit from a deeper investigation of saglin contribution during midgut infection. In addition, the manuscript would be considerably strengthened if the authors could provide direct evidence that ingested saglin promotes midgut infection.

We did try to complement *sag(-)KI* mosquitoes by feeding them with recombinant Saglin or salivary gland extracts, but this did not restore normal parasite numbers. Below, we have detailed possible reasons for this.

Reviewer #2: The work by Klug and colleagues investigates the importance of the Anopheles protein saglin for the development of Plasmodium inside its mosquito vector. Saglin supposedly acts as a receptor for parasite invasion of the mosquito’s salivary glands, a critical step to ensure transmission to a new host. The authors provide further data supporting the importance of saglin for the parasite. But their data suggest that saglin does not function as receptor during salivary gland invasion. Saglin is not expressed in those lobes of the salivary gland that are preferred by the parasites to enter the organ, which is in agreement with other cited literature.

In addition, the authors provide evidence that saglin is contained in the saliva, which is also ingested during a blood meal. Rather than being a receptor for salivary gland invasion, the presented data suggest that saglin promotes parasite development early after a blood meal.

This study is of interest for the readership of PLOS Pathogens, well-written, and the presented data are clear. Still, this manuscript may benefit restructuring to help the reader comprehending the importance of the findings. Indeed, it may be worth to consider starting the manuscript with the data on saglin (including now Fig. 8), saglin expression and localization (which support previous reports) and finishing with the new data on saglin’s pro-parasitic effect soon after the infectious blood meal.

Thank you for this concise summary of our manuscript. In the following, we have addressed the concerns and suggestions raised.

Below are specific comments, intended to further strengthen the manuscript.

Reviewer #3: This ms continues the interest in Saglin as a mosquito molecule potentially important in the infectivity of malaria parasites to mosquitoes, although studies by other groups have strongly suggested otherwise. There is an interesting conundrum presented here, that Saglin is important (but not essential) for midgut ‘colonization’ to the oocysts stage, and thereafter it has no effect at all on the conversion of oocyst to sporozoite.

The big point here also is that the effects of Saglin are observed only when the infections in mosquitoes are low. This is based on three experimental steps, each of which are slightly flawed in their approach and leave the data open to interpretation.

The effect of Saglin on mosquito infection with *Plasmodium* is actually not limited to low infections. Indeed, although there is no difference at the prevalence level, *sag(-)* knockout mosquitoes have significantly higher parasite loads at high infection densities (e.g. **Fig. 2B**). At lower densities, the difference becomes also visible for prevalence (e.g. **Fig. 2E**).

The calculation of bloodmeal size: this is done by weighing pools of mosquitoes after feeding. However, the same mosquitoes were not weighted before feeding, so it is impossible to determine what comprises final weight due to larval nutrition (resulting in increased adult body size – see Parasites & Vectors 6, 345 (2013)) or bloodmeal size. Indeed, the mosquito size after feeding seems several orders of magnitude higher (mg rather than μg) that would be expected, and the high variation in adult size from <2 mg [sic] to >5 mg suggests that rearing conditions were far from optimal.

All mosquitoes used to assess blood feeding behavior, including for the weighing experiments, were reared as a mixture of wild type and *sag(-)KI* to avoid artificial differences due to variable food quantities or splitting during larval development (lines 252-254). Because of this setup, we had not previously considered necessary to weigh unfed females. However, we agree with the reviewer that even with mixed breeding, the possibility of size bias between genotypes cannot be eliminated. Therefore, we have now included data for non-fed females, which show that *sag(-)KI* females tend to be slightly heavier than wild-type mosquitoes (**Fig. 3B**). Also, please note that the average weight of our mosquitoes before blood feeding is ~1.5mg, which is usual in our breeding conditions for *Anopheles* mosquitoes. The observed relatively high variability in weight between individual mosquitoes can be due to the fact that we bred these mosquitoes at relatively high densities.

2. The assessment of microgametocytemia is not well controlled. The blood meals were smeared onto a slide but there is no mention of how this was standardized to give a constant number of red cells read per midgut preparation. This is important when one considers the statistical approaches to sampling. When one looks at figure 3F, the presence in mosquitoes fed on highly infected mice of more insects with no microgametes in the gut is very unexpected and unexplained. Similarly, there is no mention in the methods of how high and low falciparum infections were produced. Coupled with the issues in 1, there is a real risk of multiplication of variables to give results not associated with the biology of the system. The authors should read papers by Jeff Vaughan (especially Trends Parasitol. 23:63-70 (2007)) to understand the change in dynamics of infection at different gametocytemias in the host. The fact that macrogametes are ignored in the assessments is also a cause for come concern.

We fully agree that standardization is key to this experiment and that the evaluation must be done carefully, which we have tried to do. Indeed, the data shown in Figure 3F display the percentage of exflagellated microgametes in relation to the total number of red blood cells (e.g. microgametocytemia). The confusion here has likely arisen from a mistake in the figure legend where we wrote „number of exflagellated male microgametes“ while we show the microgametocytemia in % as it was correctly indicated in the graph. We have corrected this mistake in the revised manuscript.

For each blood meal depicted in the graph, we counted the number of red blood cells in one representative field of view and the number of microgametes in 30 fields with a similar RBC density. The microgametocytemia was calculated as follows: (total number of exflagellated microgametes in 30 fields) / ((number of RBCs in one field) x 30) x 100. We have provided further details in the M&M (lines 276-279). Basically, this is similar to the procedure used to determine parasitemia in blood. We agree that smears may be uneven and that the complete blood meal may not have been spread out. However, the possible error in reading these slides should be negligible since in such cases the number of microgametes and RBCs would be affected in the same proportions.

We have considered counting the number of macrogametes in the blood meal. However, the idea of this experiment was to measure potential differences in the *sag(-)KI* genotype on the activation of gametocytes. This is relatively simple for male gametocytes since microgametes are not observed in the blood stream and result from male gametocyte activation in the mosquito, while the rupture of the RBC membrane by macrogametes is more difficult to observe. In addition, we observed that preparing a smear can lead to morphological changes in parasites without erythrocyte membrane. This is not a problem to count microgametes as they keep their characteristic shape but can lead to stronger deformations in macrogametes which may affect their counting. Macrogametes are also less abundant (PMID:30594415) increasing the risk of mistakes. We thus excluded macrogametes from the count.

As for the infections with *P. falciparum* gametocyte cultures, we have now provided some additional information concerning the concentration of gametocytes in the blood meals lines 206-207. Please note that the infectivity of *P. falciparum* gametocyte cultures can vary between cultures, which contributes to explain variation of infection parameters for a given mosquito line between different blood meals.

3. The high and low gametocytemias in the mice is unexplained. In the methods, all the mice were treated identically and yet produced different infection and different infectivities to the mosquitoes. Did the authors consider that other factors are at play here besides simple high and low microgametocytemias?

Put together, the authors could offer a more convincing case that their basic experimental design, on which all the molecular manipulations are overlaid, and the sampling and statistical assessments are robust.

In general, the methods are reasonably well written though I have offered many suggestions below. However, missing from the experimental design sections is information and detail concerning the sample sizes and replicates. These are hard to figure out later in the ms.

Indeed, we had insufficiently detailed how we produced high and low infections with *P. berghei* in the previous version of our manuscript. All infected mice were treated in the same way; the only difference between high and low infections with *P. berghei* was that mosquitoes were blood fed on mice either on day 2 (low infection) after transfer of infected blood, or on day 3 (normal/high infection) after transfer. We have now added this missing information in lines 189-191. We do not perform exflagellation assays, but track total parasitemia using flow cytometry. As we perform intravenous transfer of a precise volume of infected blood according to the parasitemia of the donor mouse, the parasitemias of recipient mice are usually uniform in all injected mice of the same batch, and reproducible between batches.

We agree with the reviewer that other factors may also influence exflagellation. However, we tried to minimize these factors by breeding *sag(-)KI* and wild-type mosquitoes as a mixture and feeding them on the same mouse. In addition, all mice were of similar age and sex, and were infected by the same procedure using the same parasite stock to keep everything standardized. Of note, we did not correct the datasets shown in **Fig. 3F & 3G** for the increased feeding capacity observed in *sag(-)KI* mosquitoes. Therefore, the observed differences in the percentage of exflagellated microgametes and in the number of basal ookinetes are likely to be even greater.

I have no comments on the genetic modifications in this ms; they are very sound and supported by strong analytical and visual data.

One major interpretation in this ms is the possible effect of Saglin as a secreted molecule being active in the midgut. In this regard the authors might have considered a simple method for assessing secreted salivary proteins (Billingsley et al. Trans RSTMH 85:450-453 (1991), and the evidence for salivary gland sporozoites and enzymes appearing in the midgut (Beier et al. AJTMH 47:195-207 (1992) and many papers by Jose Ribeiro) that actually support the physiological basis for observations.

We thank the reviewer for these suggestions. We have now included a Western blot showing that Saglin is present in large amounts in the midgut of wild-type but not *sag(-)KI* mosquitoes (**S5 Fig.**). We also experimented with forced salivation, but found this not very reliable. Therefore, we tested the presence of Saglin in the proboscis by Western blotting as a surrogate (**Figure 5D**). We have now incorporated the suggested reference Beier et al. AJTMH 47:195-207 (1992) in line 731.

---------------------------------------------

Part II – Major Issues: Key Experiments Required for Acceptance

Please use this section to detail the key new experiments or modifications of existing experiments that should be absolutely required to validate study conclusions.

Generally, there should be no more than 3 such required experiments or major modifications for a "Major Revision" recommendation. If more than 3 experiments are necessary to validate the study conclusions, then you are encouraged to recommend "Reject".

Reviewer #1: 1. The study as it stands lacks direct evidence of a role of re-ingested saliva-derived saglin in promoting Plasmodium infection in the midgut. The authors performed priming or co-feeding experiments to test this hypothesis but unfortunately no significant effect was observed in this experimental setup. The authors could attempt a more direct approach and test the effect of recombinant saglin protein supplementation in membrane feeding assays.

We agree with the reviewer that a rescue experiment with recombinant Saglin would be ideal to confirm the role of Saglin during parasite midgut invasion. However, when we initially considered this experiment, we were concerned about the functionality of the recombinant Saglin, which is derived from an *E. coli* expression system that lacks potential posttranslational modifications. Previous studies reported that Saglin is probably glycosylated at N95 (PMID: **18093000),** which we confirmed *in silico* using the NetNGlyc 1.0 prediction tool (**Fig. 1A** in this letter). In addition, we observed that recombinant and endogenous Saglin show a small but persistent size shift that could be attributed to a lack of glycosylation (**Fig. 1B,C** in this letter**)**. However, we cannot rule out the possibility that other modifications, such as phosphorylation, are required to make Saglin functional, or that the observed size shift is the result of another posttranslational modification or a combination of these. Because such a missing modification could alter Saglin function and thus its pro-parasitic activity, we were uncertain whether such an experiment would be useful (see also answer to next comment). Therefore, we first tried to circumvent the use of recombinant Saglin by testing the "priming" approach.

Based on the reviewers' comments, we have now also tested whether recombinant Saglin has a rescue effect by injecting it intravenously into mice 10 minutes before feeding. In addition, we tested whether salivary gland extracts containing endogenous Saglin could also have a rescue effect when injected into mice. Unfortunately, both approaches failed to rescue the infection phenotype in *sag(-)KI* mosquitoes. This could be due to several reasons. While the functionality of recombinant Saglin lacking post-translationsl modifications is a first concern as mentioned above, the stability of Saglin in the mouse blood circulatory system is totally unknown. There is little information in the literature about experiments in which recombinant proteins have been injected into mice to subsequently infect mosquitoes, and most experiments of this type are usually done with antibodies, which are known to be very stable and can be injected hours or even days before the infection experiment to achieve ideal distribution in the mouse. Here we kept the incubation time relatively short (10 minutes) to avoid possible degradation of the injected protein. Ultimately, this time may have been too short for the protein to reach the capillaries of the skin in significant concentration. In addition to the incubation time, the ideal concentration of Saglin is fully unknown, and salivary gland extracts obtained by dissection could only be produced in limited amounts. Because of the experimental outcome, we have now moved the data summarizing the approaches to rescue the *sag(-)KI* phenotype to the supplement (**S6 Fig.**). We describe these results in lines 632-643 and discuss possible reasons for the absence of complementation effects in lines 739-754.

Although the results of the rescue experiments are disappointing, we would like to point out that these additional experiments cement the phenotype of the *sag(-)KI* mosquito to the midgut infection phase. The Western blot now included in **S5 Fig.** shows that Saglin is present in large amounts in the midgut of wild-type mosquitoes after blood feeding, whereas it is absent in blood-fed *sag(-)KI* females.

2. The absence of saglin results in reduced oocyst burden but it is not clear at which step saglin is acting in the midgut. Is it an activation factor for male gamete formation? Is it involved in ookinete formation or migration through the midgut epithelium? Here also the authors could use the recombinant protein to test of saglin activates gametocytes, binds to ookinetes and/or promote ookinete migration.

We fully agree with the reviewer that deciphering the role of Saglin in promoting parasite development in the midgut is of great interest, although beyond the scope of the study presented here. Still, as discussed above and shown in **S6 Fig.**, we could not rescue the *sag(-)* phenotype by supplementation with recombinant Saglin., suggesting that it is non-functional. Obviously, it would be possible to produce it in insect cells to test again whether it could rescue the *sag(-)KI* phenotype and, should it do, investigate which steps of parasite development are boosted by Saglin.

**Fig. 1. Potential post-transcriptional modification of Saglin**

**A)** The first 64 amino acids after the predicted cleavage site of the signal peptide are shown. Based on predictions using NetNGlyc 1.0, N95 is presumably glycosylated. The terminal **-C** represents the entire remaining C-terminus of the protein. **B)** Western blot of recombinant Saglin expressed in *E. coli* adjacent to endogenous Saglin from 10 dissected salivary glands (SGs). Note the small size shift between signals. The upper and lower bands correspond to the sizes of a dimer and a monomer of Saglin, respectively. **C)** Western blot of different tissues of a mosquito strain expressing *DsRed* in the distal-lateral lobes driven by the *aapp* promoter (*aapp-DsRed*) (PMID: 36223382). Salivary glands were separated into samples containing both median and proximal-lateral lobes and distal-lateral lobes to test the accuracy of the dissections by detecting Saglin (expressed in the median lobe) and DsRed (expressed in the distal-lateral lobes). In addition, hemolymph and a carcass sample without salivary glands were loaded alongside recombinant Saglin to test for possible secretion of Saglin in hemolymph and the presence of Saglin in other tissues. The dashed red lines indicate the midpoint of the signals from recombinant and endogenous Saglin monomers to better visualize the difference in size.

Reviewer #2: (No Response)

Reviewer #3: (No Response)

---------------------------------------------

Part III – Minor Issues: Editorial and Data Presentation Modifications

Please use this section for editorial suggestions as well as relatively minor modifications of existing data that would enhance clarity.

Reviewer #1: -line 29: unicellular

Corrected.

-line 109-110: SM1 is a mimotope of TRAP and binds to saglin not to TRAP

The sentence was rephrased (lines 118-123).

-throughout the manuscript the authors sometimes use “distal” to refer to “distal lateral” or “lateral” when describing salivary gland lobes.

As suggested, we have harmonized the nomenclature throughout the revised manuscript.

-In Fig2 (and others) the graphs show median values, while the text refers to mean values. It might be useful to

harmonize the description of the data between figures and text.

We have carefully reviewed all the graphs and indicated clearly whether they show medians or means, and which statistical test was used for each.

-Line 430: Fig2 supplt 1 instead of Fig 2C

Corrected.

-Line 494: mosquitoes were

Corrected.

-Lines 674-678: the authors could discuss the possible reasons why the results are discordant with the Ghosh et al study. Do they envisage that TRAP-saglin interaction could still play a role in the salivary glands for transmission?

Indeed, we cannot rule out that Saglin may also support the transmissibility of sporozoites, as reported for TRIO. To point this out we have added now the sentence „Therefore, it cannot be excluded that Saglin has a stimulatory effect on sporozoites in the skin and increases their transmissibility.“ (lines 716-718).

-Fig2, 3, 5, 6, 7, 2S1, 5S1: please carefully check the figures, frames are visible around some of the panels.

The images in all figures are intentionally framed by black squares to increase the contrast between the image and the background. Other than that, we could not see any frames in the figures mentioned. If we missed something, we would be grateful if the reviewer could describe in more detail which panels are framed.

-line 929: why showing five countings instead of the three values for the independent experiments?

We now display the mean of each experiment in this dataset, as suggested.

-line 930: the standard error is not shown in fig2C

There was a mistake in the legend. There is actually no standard error shown and the blue line indicates now the mean. We have corrected the passage accordingly.

-line 988: DL and PL likely refer to distal lateral and proximal lateral, respectively

This is right. We have corrected the mistake in the revised manuscript.

Reviewer #2: A rescue experiment would certainly provide robust support for the hypothesis that “saglin ingested with the blood meal may have a pro-parasitic effect on Plasmodium midgut colonization”. But the conducted experiment (Fig. 6) seems not well-fitted to test this hypothesis. Especially since the presence of WT saliva (including saglin) at the bite site cannot be controlled for. Also do mosquitoes use the same bit site at all? A better rescue experiment may be to supplement saglin in a membrane feed into sag(-)KI mosquitoes. Thus, although it is formally not wrong, Fig. 6 and the respective text is not convincing as these experiments do not answer the question whether saglin supplementation can rescue parasite development in sag(-)KI mosquitoes. Please revise.

As described above, we have now performed additional experiments aiming to rescue the *sag(-)KI* phenotype in different ways, unfortunately without success. See above and S6 Fig..

The experiment leading to the data shown in Fig. 5B is unclear. Please explain what XFD594 is and, more importantly, how WGA injection helps answering the question if sporozoites can access saglin coming from the hemolymph. Currently, the significance of this experiments and thus the conclusions of the WGA staining are unclear.

XFD594 is a fluorescent dye and a structural homolog to Alexa Fluor® 594. We have now inserted "...structural analogue of Alexa Fluor® 594..." in line 391. We hypothesized that although Saglin promoter activity was observed only in the median lobe, the protein might be secreted into the hemolymph to act as a chemoattractant for sporozoites to find the salivary glands, or that the median lobe might serve as an entry point from which sporozoites translocate to the lateral lobes. To assess this idea, we used fluorescently labeled WGA to stain the surface of the gland *in vivo* and test whether the median lobe makes contact with the hemolymph, which would be beneficial, if not necessary, for secretion and invasion. We have tried to describe our working hypothesis and experimental setup more clearly in lines 589-595, and we refer to the results of these experiments in discussion lines 701-703.

L 47: Please explain the abbreviation DENV as you are using it later in line 51.

We added „Dengue virus, DENV“ in line 65.

L 77 and throughout the manuscript: To distinguish Anopheles and Aedes mosquitoes, I thought Anopheles is abbreviated An. Please edit accordingly, especially since Aedes aegypti in line 654 is also abbreviated as “A.” Also, it is unclear why Anopheles is sometimes abbreviated and sometimes not. Please harmonize.

We have followed the advice of the reviewer. „*Anopheles*“ is now abbreviated as „*An.*“ throughout the manuscript, and *Aedes* as *Ae*.

L98: What is a one to one orthologue? Is it highly conserved?

A one-to-one orthologue is a gene that is indeed highly conserved in two or more species. Gene 1 of species 1 has a one to one orthologue in species 2 when blasting gene 1 against the genome of species 2 will give gene 2 as a single main hit, and reciprocally, blasting gene 2 against the genome of the species 1 will detect the gene 1 (the original query) as a single main hit.

Still, to make it clearer, we modified the sentence. It reads now „...CSP-BP, which is highly conserved across insects and corresponds to UPF3 in *Drosophila*.”.

L 106-109: The discussion would benefit from mentioning the injected anti saglin mAB work again and how these results fit to new proposed function of saglin.

As suggested, we have extended the presentation of the results that led to the model where Saglin could act as a salivary gland receptor for sporozoites (injection/ingestion of anti-Saglin antibody and SM1 peptide limiting sporozoite numbers in the salivary glands) in a new paragraph (lines 689-696).

L 109: Please provide more information on the circular peptide SM1. Where does it come from?

See answer to point just above

L 177: Should it be 2 x 10^7 rather than 2.1^7?

Thank you for spotting this error. We have corrected the mistake.

L 420 and following: It is confusing to report the mean in the text and the median in the figure. Although not wrong, please consider revising this.

Similarly, in the text wild-type mosquitoes are described as control, but in the Fig. wild-type is used as label.

We have carefully reviewed the figures and figure legends to make sure that they are coherent concerning medians and means. We have also normalized the nomenclature and use now „wild-type“ throughout the manuscript.

L430: Fig. 2C shows the number of SGS not the number of oocysts. Please revise.

Corrected.

Caption of Fig. 2E: Please add that the red bars indicate.

The red bar indicates the median in this experiment. We have added the missing information in the figure legend (lines 1.003-1.004).

A panel “F” is not part of Fig. 2 and the respective caption. Please revise.

Corrected.

L 463: “Plasmodia” is not a taxonomical term and should neither be italicised nor capitalized. Also, it has been critically discussed in the literature (Plasmodia - don’t by McFadden, 2012). Please change.

Thank you for this interesting comment. We have changed „*Plasmodia*“ to „*Plasmodium spp.*“.

Throughout the manuscript: Please consistently use either wild type or WT, see e.g., in lines 466 and 469.

Thank you for spotting this inconsistency. We now use „wild-type“ throughout the revised manuscript.

L 473 and 474: Please provide a reference for this statement or delete.

We have now provided a reference for this statement (PMID: 1770513).

Fig. 3E: Could you use a scale bare that has not the approximate length of 7 µm? Also, in the other figures, the length of the scale bar is provided in the caption rather than the image itself.

The images shown in Fig. 3E were taken with an IPhone through the lens of a light microscope without a proper holder to define a precise distance. Therefore it is not possible to calculate an exact scale for these images. We indicated a scale of ~7 µm because this is the average diameter of a mouse red blood cell. However, we have decided now to remove the scale completely. Instead we have included the following sentence in the legend of Fig. 3 :“ Due to the recording method, it was not possible to draw a precise scale. As a rough estimate, the diameter of a red blood cell can be assumed to be 7 µm.”.

L 548: Here a little more context would be helpful. To my knowledge sporozoites prefer the lateral lobes, which could be mentioned in this sentence. And you show that saglin not expressed in the lateral lobes. Just found this information much below (line 567), including a reference. Perhaps consider providing this information already here.

We have adopted the reviewer's suggestion and moved the passage in question (line 572).

L 564: As there is no statistical analysis, the term “significantly” should be revised.

Corrected.

L 589: Please explain what proboscis samples are. Is this saliva or the actual organ?

This is the complete organ. We have clarified the wording in the revised manuscript (e.g. line 613). The procedure for making the samples is explained in detail in lines 333-335.

Paragraph starting in line 618 does not reference Fig. 7B. Please revise. Also, the data shown in Fig. 7 are related to those in Figs. 2 and 3 and it is unclear why these are separated (see also comment above).

Thank you for pointing out this discrepancy. We have added a reference to Fig 7 in the corresponding paragraph of the revised manuscript.

We also agree that the data shown in **Fig. 7** are related to the differences in infection densities shown in **Fig. 2** and **Fig. 3**. However, we do not feel it is necessary to directly link these data in the same figure because we address mosquito infection and parasite transmission from mosquito to mouse separately.

L696: “regurgitate” does not seem to make sense in this context. Should it have been re-ingest?

The sentence now reads „re-ingest“ as suggested.

Fig. 8 and the corresponding text in line 732-736 should be incorporated in the results section, perhaps as new Fig. 1.

Thank you very much for this suggestion. We have now incoprorated some of the sequence data in Fig. 1 and reference the alignment now earlier in the manuscript (line 132 ).

Reviewer #3: Specific comments (by line number):

59 The red cell membrane is ruptured rather than shed.

We have modified the sentence accordingly. It reads now „...leading to the rupture of the red cell membrane upon activation” (lines 72-73).

63 The language is imprecise here. The ookinetes traverse the midgut epithelial cells, not the whole epithelium. The ookinetes rest upon the basal lamina not below it. The cells confer a polarity to the basal lamina, and the ookinetes rest on it and facing the haemocoele but still separated from it by the basal lamina.

Thank you for pointing this out. We have changed the wording accordingly (lines 76-77).

66 The ookinete is not half-moon shaped (that would be a semi-circle).

We have changed " half-moon" to "crescent” -shaped sporozoites" on line 78.

69 Should read kinetics.

Corrected.

70 Most of the introduction to this point is superfluous – does not contribute to the core of the ms.

We have shortened the introduction to make it more concise. Still we have retained a brief summary of the *Plasmodium* life cycle as we feel this is necessary to keep the conclusions and experiments performed understandable to researchers not working directly with *Plasmodium*.

135 Is the key resources table required by the journal? It seems an odd inclusion. It does not have a legend and references should go back to source (for example NF54 was generated in The Netherlands many years prior to Bryant et al. 2018).

As we understand the authoring guidelines, the key resources table is required. For the reference Bryant et al. 2018, please see comment on line 185 below.

139-42 The mosquito strains here do not appear in the resources table

We thank the reviewer for pointing out this error to us. Both missing mosquito strains appear now in the key resources table.

148 and elsewhere – “h” is the correct abbreviation for hours

We have replaced „hours“ with „h“ throughout the manuscript.

149 “Osmosed” is not correct

We have changed „Osmosed“ to „purified by osmosis“.

154 why “floated”?

This is the term we normally use in the lab for this process. We have simplified the passage (lines 158-165).

160 change “neonate” (a term describing a mammalian baby) to “first instar”

We have followed the suggestion of the reviewer and modified the sentence accordingly.

172 Again, precision – the mice were injected with thawed P. berghei blood stages in mouse blood….how many parasites per mouse? How controlled were these infections? Were mice treated beforehand with phenylhydrazine as done in all Sinden papers?

The method was actually precisely detailed (lines 179-194).

We use a highly reliable protocol to achieve very consistent *P. berghei* infections by performing fresh blood transfers from an infected mouse into naive mice by intravenous transfer of 2 x 10^7^ parasites. Only the donor mouse is injected with a frozen parasite stock. We have now added information on the production of these stocks and an estimate of the number of parasites they contain (lines 192-194). However, this information is not directly relevant to subsequent infection because, due to parasite loss during freezing and differences in injection procedure (depth and location of intraperitoneal injection differ between experimenters), these donor mice exhibit some variability in how quickly parasites appear in the bloodstream. To avoid artefacts linked to this variability, we transfer the fresh infected blood from this donor mouse to a different batch of mice that will be used for mosquito infections. By transferring blood intravenously in recipient mice, we obtain much more uniform parasite growth in the different mice used for infection, which we also monitor closely using flow cyometry. In our experience, this procedure is much more precise than intraperitoneal transfer of fresh blood, which results in a wider variation of parasitemia in recipient mice.

We do not perform phenylhydrazine injections because, in our experience, mosquito infections are not improved with this approach. In addition, it has been shown that there is a negative correlation between high gametocyte densities and oocyst burden in *P. berghei* (PMID: 25541384). Phenylhydrazine injections are especially useful when gametocytes are to be studied or for culturing ookinetes *in vitro*.

179 delete “when”

According to our spelling correction programs, "when" is necessary at this position. However, we have now removed the comma in front of it.

185 Bryant is an inappropriate reference here

Different *Plasmodium* clones, even when derived from the same isolate, can exhibit a wide variety of phenotypes, as has been shown for drug sensitivity (PMID: 2091341). To make our experiments as reproducible as possible, we used the NF54 clone A11 reported and sequenced by Bryant et al. (2018). Therefore, we believe that this reference is appropriate here. However, we have included a second reference on the origin of the NF54 background (PMID: 3299700).

189-90 Number “2” should be subscripted in chemical formulae

Corrected.

195 parafilm should be Parafilm™

Corrected.

203 Usually berghei oocysts are counted on day 10, falciparum on day 7-8

*P. berghei* oocysts can be counted at a later time, but they are already very easily counted at d7-8 post infection. Keeping them 2-3 days more before dissection would lead to bigger oocysts, but we never observed major changes in prevalences and parasite numbers during this period.

208 replace ,, with “

We replaced „with“ by „by“.

212 Oocysts were manually counted using software? I don’t understand.

We have now specified what we meant by adding the phrase „...manually using the “multi-point” tool of Fiji.”

215 Ground not grinded; here and throughout ms, use decimal point rather than comma (1.5 mL)

We have corrected both errors throughout the manuscript.

219 Why such low magnification? There would be a lot of detritus here which could easily obfuscate the counting

The next higher magnification of the microscope we use is 40x, which is an oil objective. Since the glass cover of the counting chamber cannot be sealed, the pressure of the objective on the slide sometimes causes a slight displacement of the cover and turbulence in the sample leading to inaccurate counts. Therefore, we prefer to use the 20x objective, which provides a good overview, while the sporozoites can still be seen and counted very well in darkfield. We usually do not have problems with detritus. When salivary glands are dissected without additional tissue, diluted samples appear almost completely detritus-free.

245-6 Not clear if individuals were weighed or pools

We have rephrased this paragraph to be more precise (line 258 and line 260). Blood fed females were weighed individually while for sugar fed controls we have decided to weigh pools of 25-50 mosquitoes of each genotype. Both weighing protocols indicate that *sag(-)KI* females are slightly heavier compared to wild-type.

247 Their abdomens “were”…

Corrected.

249 7 μl “were” loaded (please check throughout). How did the authors control for rupture of red cells using this method?

Note – I have stopped going through the methods line-by-line here – it is too much work for a reviewer

We have corrected this spelling error throughout the manuscript.

We have not controlled for the rupture of red blood cells. Of note, samples were taken very shortly after feeding (10 minutes) when the blood homeostasis was still intact. We could not observe any morphological changes by microscopy in the erythrocytes such as echinocytosis at this time point. We also did not observe any cellular debris or erythrocyte "ghosts" that might indicate lysis. Nevertheless, we cannot exclude the possibility that some lysis had already occurred, but we believe that this would be the case to a similar extent in all samples.

We thank the reviewer for taking the time to review the methods carefully. We have further revised the entire M&M to correct mistakes and make it more precise.
