# Supplementary material for: The salivary protein Saglin facilitates efficient midgut colonization of Anopheles mosquitoes by malaria parasites
Source: PLoS Pathog. 2023 Mar 2;19(3):e1010538. doi: 10.1371/journal.ppat.1010538 (PMC10013899; doi:10.1371/journal.ppat.1010538)
Supplement: Attachment — Submitted filename: PPATHOGENS-D-22-00735_rebuttal_letter_260123.docx [file ppat.1010538.s007.docx]

Marburg, January 26th 2023

To the editorial board,

PLOS Pathogens

**Dear Editorial Board,**

We would like to thank you and the reviewers for the evaluation of our manuscript entitled “The salivary protein Saglin facilitates efficient midgut colonization of *Anopheles* mosquitoes by malaria parasites”. We are grateful to the reviewers’ positive feedback and for the very good comments and suggestions. We have revised our manuscript accordingly, and hope you will consider it suitable for publication.

With our best wishes for 2023,

Dennis Klug & Stéphanie Blandin

Dear Mr. Klug,

Thank you very much for submitting your manuscript "The salivary protein Saglin facilitates efficient midgut colonization of Anopheles mosquitoes by malaria parasites" for consideration at PLOS Pathogens. As with all papers reviewed by the journal, your manuscript was reviewed by members of the editorial board and by several independent reviewers. The reviewers appreciated the attention to an important topic. Based on the reviews, we are likely to accept this manuscript for publication, providing that you modify the manuscript according to the review recommendations.

Please prepare and submit your revised manuscript within 30 days. If you anticipate any delay, please let us know the expected resubmission date by replying to this email.

When you are ready to resubmit, please upload the following:

[1] A letter containing a detailed list of your responses to all review comments, and a description of the changes you have made in the manuscript.

Please note while forming your response, if your article is accepted, you may have the opportunity to make the peer review history publicly available. The record will include editor decision letters (with reviews) and your responses to reviewer comments. If eligible, we will contact you to opt in or out

[2] Two versions of the revised manuscript: one with either highlights or tracked changes denoting where the text has been changed; the other a clean version (uploaded as the manuscript file).

Important additional instructions are given below your reviewer comments.

Thank you again for your submission to our journal. We hope that our editorial process has been constructive so far, and we welcome your feedback at any time. Please don't hesitate to contact us if you have any questions or comments.

Sincerely,

Oliver Billker

Academic Editor

PLOS Pathogens

Kirk Deitsch

Section Editor

PLOS Pathogens

Kasturi Haldar

Editor-in-Chief

PLOS Pathogens

orcid.org/0000-0001-5065-158X

Michael Malim

Editor-in-Chief

PLOS Pathogens

orcid.org/0000-0002-7699-2064

***********************

Reviewer Comments (if any, and for reference):

Reviewer's Responses to Questions

Part I - Summary

Please use this section to discuss strengths/weaknesses of study, novelty/significance, general execution and scholarship.

Reviewer #1: (No Response)

Reviewer #3: The authors have undertaken a lot of work to address what were substantial comments from three referees. While some of the results are still not conclusive, further work would be outside the scope of this manuscript.

We would like to thank reviewers 1 and 3 for the time they took to re-evaluate our manuscript and for acknowledging the work we put into the revision. Below we have explained how we addressed the remaining comments.

---------------------------------------------

Part II – Major Issues: Key Experiments Required for Acceptance

Please use this section to detail the key new experiments or modifications of existing experiments that should be absolutely required to validate study conclusions.

Generally, there should be no more than 3 such required experiments or major modifications for a "Major Revision" recommendation. If more than 3 experiments are necessary to validate the study conclusions, then you are encouraged to recommend "Reject".

Reviewer #1: (No Response)

Reviewer #3: None.

---------------------------------------------

Part III – Minor Issues: Editorial and Data Presentation Modifications

Please use this section for editorial suggestions as well as relatively minor modifications of existing data that would enhance clarity.

Reviewer #1: The authors have modified the text to better reflect the existing data. While it is unfortunate that they did not attempt the rescue experiment through membrane feeding (which would probably be more efficient than injection into mice), they do refer to this experiment in the discussion. It will be interesting to know the outcome of such an experiment, which could bring some novel insights into the role of saglin during Plasmodium infection.

I only have a few minor comments:

1) Fig1B could be cited line 130

Done.

2) Fig3C shows no difference in weight after blood meal, therefore the statement line 502-503 should be modified.

Thank you for pointing this out. We modified the sentence to describe the results more precisely. It reads now „Accordingly, *sag(-)KI* females tended to have higher weight after blood feeding than wild-type females, although this difference was not significant.“ (lines 503-505).

3) The western blot in fig S5 is intriguing, as there is much more saglin detected in 5 midgut samples as compared to 8 salivary glands. It looks like there is a massive protein band (presumably albumin) in the WT lane that could interfere with saglin detection. For some reason this interfering protein accumulation is not seen (or not to the same extent) in the KO lane. I suggest removing this figure. The information that saglin is detected in the midgut of blood-fed mosquitoes is already shown in fig 5F.

As suggested by the reviewer, this figure is not essential to demonstrate that Saglin is present in the midgut of blood-fed mosquitoes, and we have therefore removed Fig S5 from the revised manuscript to avoid any confusion.

Still, we would like to provide some explanation to the Western Blot shown in Fig S5. It is indeed correct that the Saglin band is much more intense in the 5 midguts than in the 8 salivary glands, all dissected from the same 5 mosquitoes maximum 10 min after a blood feeding. In this case, the strong difference in signal can be explained by the following facts, ranked by importance: (1) Saglin that had been accumulated in the salivary glands of these 5 mosquitoes, was injected in the skin of the mouse together with saliva and re-ingested during the blood meal, hence the observed depletion of Saglin in the salivary glands, and the inverse accumulation of Saglin in the midgut. We have observed this clear depletion of Saglin in the salivary glands of mosquitoes after blood feeding in several western blots, albeit not always with the same intensity. (2) Two salivary glands are missing in the SG sample as compared to the number of midguts. And finally, (3) as noted by the reviewer, migration of the midgut sample was somehow distorted (probably due to the large concentration of proteins), which may also have affected the signal in the salivary gland sample.

Reviewer #3: (No Response)

---------------------------------------------

PLOS authors have the option to publish the peer review history of their article (what does this mean? [https://journals.plos.org/plospathogens/s/editorial-and-peer-review-process#loc-peer-review-history](https://journals.plos.org/plospathogens/s/editorial-and-peer-review-process#loc-peer-review-history)). If published, this will include your full peer review and any attached files.

If you choose “no”, your identity will remain anonymous but your review may still be made public.

Do you want your identity to be public for this peer review? For information about this choice, including consent withdrawal, please see our Privacy Policy [https://www.plos.org/privacy-policy](https://www.plos.org/privacy-policy).

Reviewer #1: No

Reviewer #3: Yes: Peter F Billingsley
